# *Salmonella enterica* serovar Typhimurium *sseK3* induces apoptosis and enhances glycolysis in macrophages

**DOI:** 10.1186/s12866-020-01838-z

**Published:** 2020-06-09

**Authors:** Chuan Yu, Fuyu Du, Chunjie Zhang, Yinju Li, Chengshui Liao, Lei He, Xiangchao Cheng, Xiaojie Zhang

**Affiliations:** 1 The Key Lab of Animal Disease and Public Health, Henan University of Science and Technology, 263 Kaiyuan Avenue, Luoyang, 471023 Henan China; 2 Luoyang Key Laboratory of Live Carrier Biomaterial and Animal Disease Prevention and Control, Luoyang, 471023 Henan China; 3 Luoyang Polytechnic, 6 Airport Road, Luoyang, 471023 Henan China

**Keywords:** *S.* Typhimurium, *sseK3*, Macrophages apoptosis, Glycolysis

## Abstract

**Background:**

*Salmonella enterica* serovar Typhimurium (*S.* Typhimurium) is an important infectious disease pathogen that can survive and replicate in macrophages. Glycolysis is essential for immune responses against *S.* Typhimurium infection in macrophages, and is also associated with apoptosis. *S.* Typhimurium secreted effector K3 (SseK3) was recently identified as a novel translated and secreted protein. However, there is no study about the role of *sseK3* in the relationship between apoptosis and glycolysis in cells infected with *S.* Typhimurium. It is unclear whether this protein exerts a significant role in the progress of apoptosis and glycolysis in *S.* Typhimurium-infected macrophages.

**Results:**

Macrophages were infected with *S.* Typhimurium SL1344 wild-type (WT), Δ*sseK3* mutant or *sseK3*-complemented strain, and the effects of *sseK3* on apoptosis and glycolysis were determined. The adherence and invasion in the Δ*sseK3* mutant group were similar to that in the WT and *sseK3*-complemented groups, indicating that SseK3 was not essential for the adherence and invasion of *S.* Typhimurium in macrophages. However, the percentage of apoptosis in the Δ*sseK3* mutant group was much lower than that in the WT and *sseK3*-complemented groups. Caspase-3, caspase-8, and caspase-9 enzyme activity in the Δ*sseK3* mutant group were significantly lower than in the WT group and *sseK3*-complemented groups, indicating that *sseK3* could improve the caspase-3, caspase-8, and caspase-9 enzyme activity. We also found that there were no significant differences in pyruvic acid levels between the three groups, but the lactic acid level in the Δ*sseK3* mutant group was much lower than that in the WT and *sseK3*-complemented groups. The ATP levels in the Δ*sseK3* mutant group were remarkably higher than those in the WT and *sseK3*-complemented groups. These indicated that the *sseK3* enhanced the level of glycolysis in macrophages infected by *S.* Typhimurium.

**Conclusions:**

*S.* Typhimurium *sseK3* is likely involved in promoting macrophage apoptosis and modulating glycolysis in macrophages. Our results could improve our understanding of the relationship between apoptosis and glycolysis in macrophages induced by *S.* Typhimurium *sseK3*.

## Background

*Salmonella enterica* serovar Typhimurium (*S.* Typhimurium) is a zoonotic pathogen that can infect humans [1]. *Salmonella* can be transmitted to humans via contaminated animal products, causing illness and potentially deaths [1]. *S.* Typhimurium can survive and replicate in macrophages, which could carry bacteria from the Peyer’s patches to adjacent lymph nodes, the spleen and the liver in mouse models [2].

Glycolysis is an essential cellular metabolic pathway [3]. Under anaerobic conditions, the pyruvate is eventually converted into lactic acid. However, under aerobic conditions, pyruvic acid enters the tricarboxylic acid cycle (TCA cycle) and is oxidized to CO_2_ and H_2_O [4]. Apoptosis is closely associated with glycolysis activities [5, 6]. Inhibition of the glycolysis with iodoacetate was associated with macrophage apoptosis [7], and genetic silencing of hypoxia inducible factor-1α (HIF-1α) repressed imiquimod-induced aerobic glycolysis and sensitized cells to imiquimod-induced apoptosis owing to faster ATP and Mcl-1 depletion [5]. Sirtuin 6 modulates hypoxia-induced apoptosis in osteoblasts via inhibition of glycolysis, and hypoxia-induced apoptosis of osteoblasts is dependent on glycolytic activity [8]. Several molecules that function intracellularly as enzymes are involved in glycolysis, and become externalized to the surface of apoptotic cells, causing apoptotic recognition and the triggering innate apoptotic immunity [9]. Thus, glycolysis is closely related to apoptosis.

Immune cells were able to detect the metabolic abnormalities caused by *Salmonella* through inflammatory signals, and glycolysis was essential for this process in *S.* Typhimurium-infected with macrophages [10, 11]. Salmonella pathogenicity island 2 (SPI-2)-encoded type III secretion system 2 (T3SS2), which delivers 28 effector proteins into the host cell, is one of the key virulence determinants after invasion or phagocytic uptake of *Salmonella* [12–14]. Previous studies have identified that SseK3 is a novel translated and secreted protein of *S.* Typhimurium, and is encoded by the *sseK3* gene [15]. SseK3 is a glycosyltransferase, and could transfer an N-acetyl-glucosamine moiety onto the guanidino group of a target arginine, thereby regulating host cell function [16]. It belongs to the glycosyltransferase type-A family of glycosyltransferase enzymes and binds ligands in a metal-ion-dependent manner via a DXD motif [16]. SseK3 is co-regulated with the T3SS2 in host cells and is injected into infected host cells [17]. However, the mechanisms underlying SseK3 activity during S. Typhimurium infection and the role of *sseK3* in macrophage glycolysis remain unclear.

In this study, we aimed to determine the role of *S.* Typhimurium *sseK3* on macrophage apoptosis and glycolysis after *S.* Typhimurium infection. Our data showed that the *sseK3* of *S.* Typhimurium could promote macrophage apoptosis and improve glycolysis levels. These results would provide a better understanding of the relationship between glycolysis and apoptosis in *S.* Typhimurium-infected macrophages.

## Results

### Adherence and invasion

The invasive and adhesive abilities of the *sseK3* mutant and complemented strain were similar to those of the WT strain (Fig. S1A, Fig. S1B); there were no significant differences between the WT, Δ*sseK3* mutant, and *sseK3*-complemented groups (*P* > 0.05). This suggests that *sseK3* does not play a prominent role in promoting the attachment and invasion of *S.* Typhimurium into the macrophages. Because the same number of bacteria invaded macrophages of the different infection groups, the intracellular load was consistent, and improved the accuracy of subsequent experiments.

### Apoptosis

**Fig. 1:**
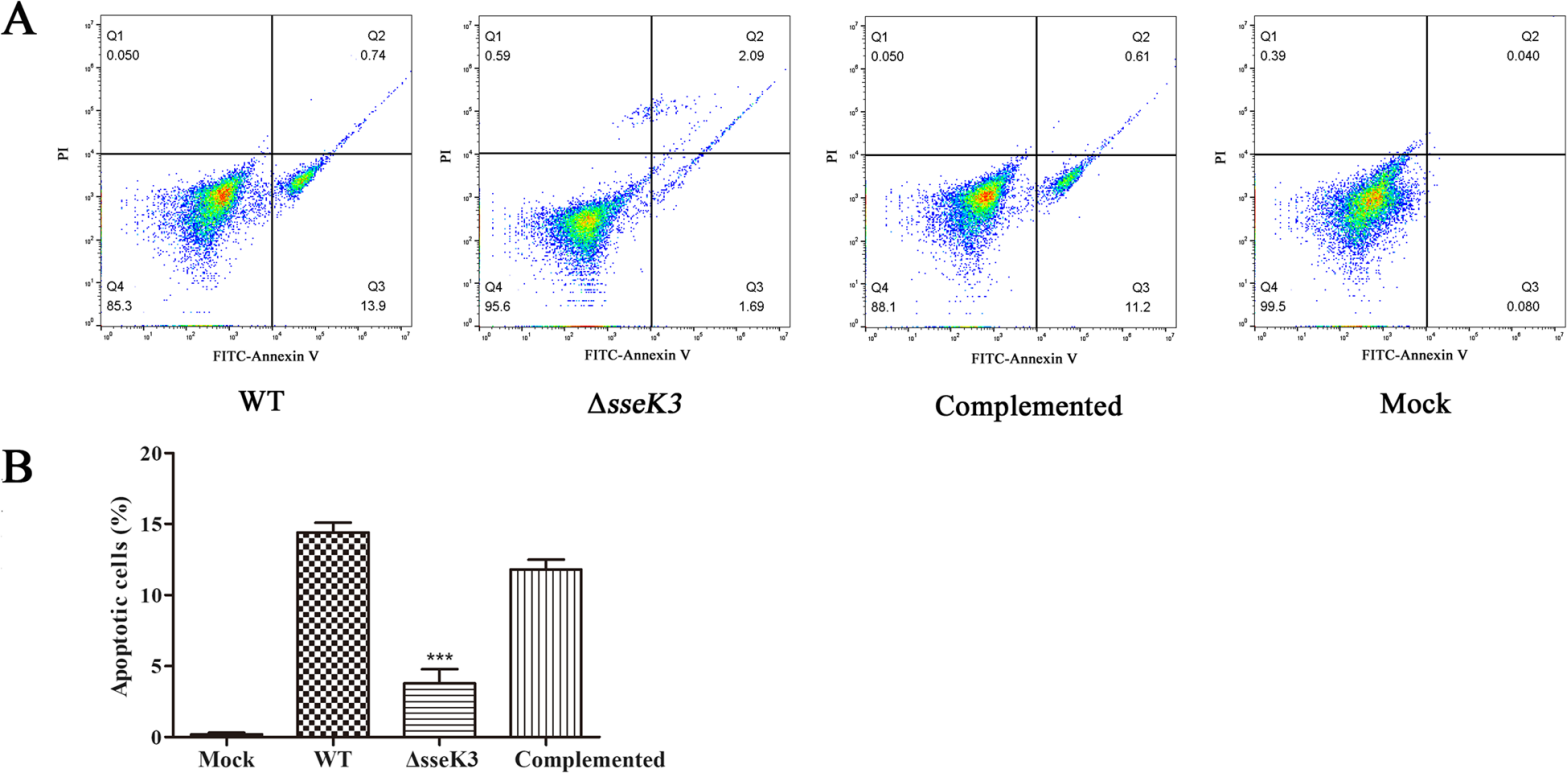
Flow cytometric analysis of apoptosis in RAW264.1 macrophages infected with WT *S.* Typhimurium, Δ*sseK3* mutant, or complemented strains. RAW264.1 macrophages were incubated for 4 h with WT *S.* Typhimurium, Δ*sseK3* mutant, or *sseK3* complemented strains. Apoptosis was assessed using Annexin V-FITC/PI double staining. (**a**) Representative dot plots showing Annexin V-FITC /PI staining. The upper left quadrant (Q1) contains the necrotic (Annexin V-FITC−/PI+) population, the upper right quadrant (Q2) contains the late apoptotic (Annexin V-FITC+/PI+) population, and the lower right quadrant (Q3) contains the early apoptotic (Annexin V-FITC+/PI-) population. The lower left quadrant (Q4) contains the healthy (double negative) population. (**b**) Percentage of apoptosis in cells exposed to different bacteria determined by flow cytometric analysis. After 4 h of incubation, the apoptosis rate in the Δ*sseK3* mutant group was significantly lower than that in the WT and *sseK3*-complemented groups (***P < 0.001). Data from three independent experiments were used to determine percentage of apoptotic cells. Error bars indicate the SD from three independent experiments

Apoptosis assays were performed as previously described [18, 19]. The percentage of apoptotic cells of Δ*sseK3* mutant group was much lower than that in the WT and *sseK3*-complemented groups. In the Δ*sseK3* mutant group, the percentage of FITC-Annexin V positive cells was 3.78%, while in the WT group, it was 14.64% (Fig. 1), suggesting that SseK3 plays an important role in *S.* Typhimurium-induced macrophage apoptosis.

### Caspase activity

**Fig. 2:**
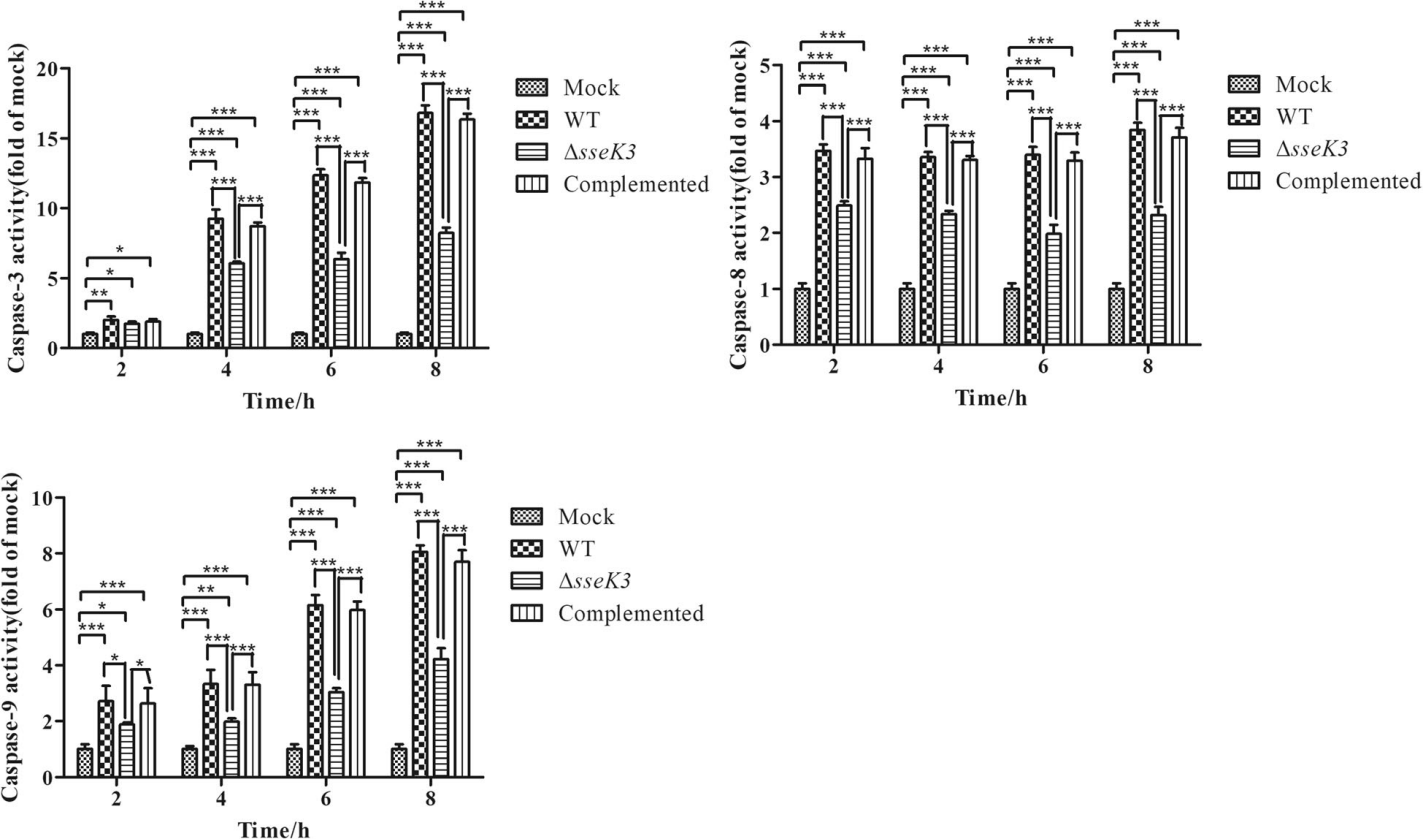
Caspase-3, caspase-8, and caspase-9 activity in RAW264.7 cells infected with WT *S.* Typhimurium, Δ*sseK3* mutant, or *sseK3-*complemented strains. Caspase-3, − 8, and − 9 activity was measured by colorimetric assay and analyzed using two-way ANOVA with Bonferroni’s multiple-comparison test (*P < 0.05,**P < 0.01,***P < 0.001). Results are shown as mean ± SD

Caspases play an essential role in apoptosis [20]. Caspase-3, 8, and 9 activity was therefore measured at different time points (Fig. 2), and was much lower in the mock group than in the infection groups (Δ*sseK3* mutant, WT, and *sseK3*-complemented groups), indicating that *S.* Typhimurium infection could stimulate the activity of caspase-3, 8, and 9 in macrophages. However, it could be seen from Fig. 2 that caspase-8 and caspase-9 activity was significantly lower in the Δ*sseK3* mutant group than that in the WT and *sseK3-*complemented groups at 2 h, 4 h, 6 h, and 8 h (*P* < 0.001), suggesting that SseK3 could induce the activation of caspase-8 and caspase-9. There was no significant difference in caspase-3 activity among the Δ*sseK3* mutant, WT, and *sseK3-*complemented groups at 2 h (*P* > 0.05). However, significant differences in caspase-3 activity were observed among the infection groups at 4 h, 6 h, and 8 h (*P* < 0.001). Caspase-3 activity was significantly lower in the Δ*sseK3* mutant group than in the WT and *sseK3-*complemented groups. These results illustrated that SseK3 likely played a pivotal role in the process of inducing macrophage apoptosis by *S.* Typhimurium.

### Glycolysis

**Fig. 3:**
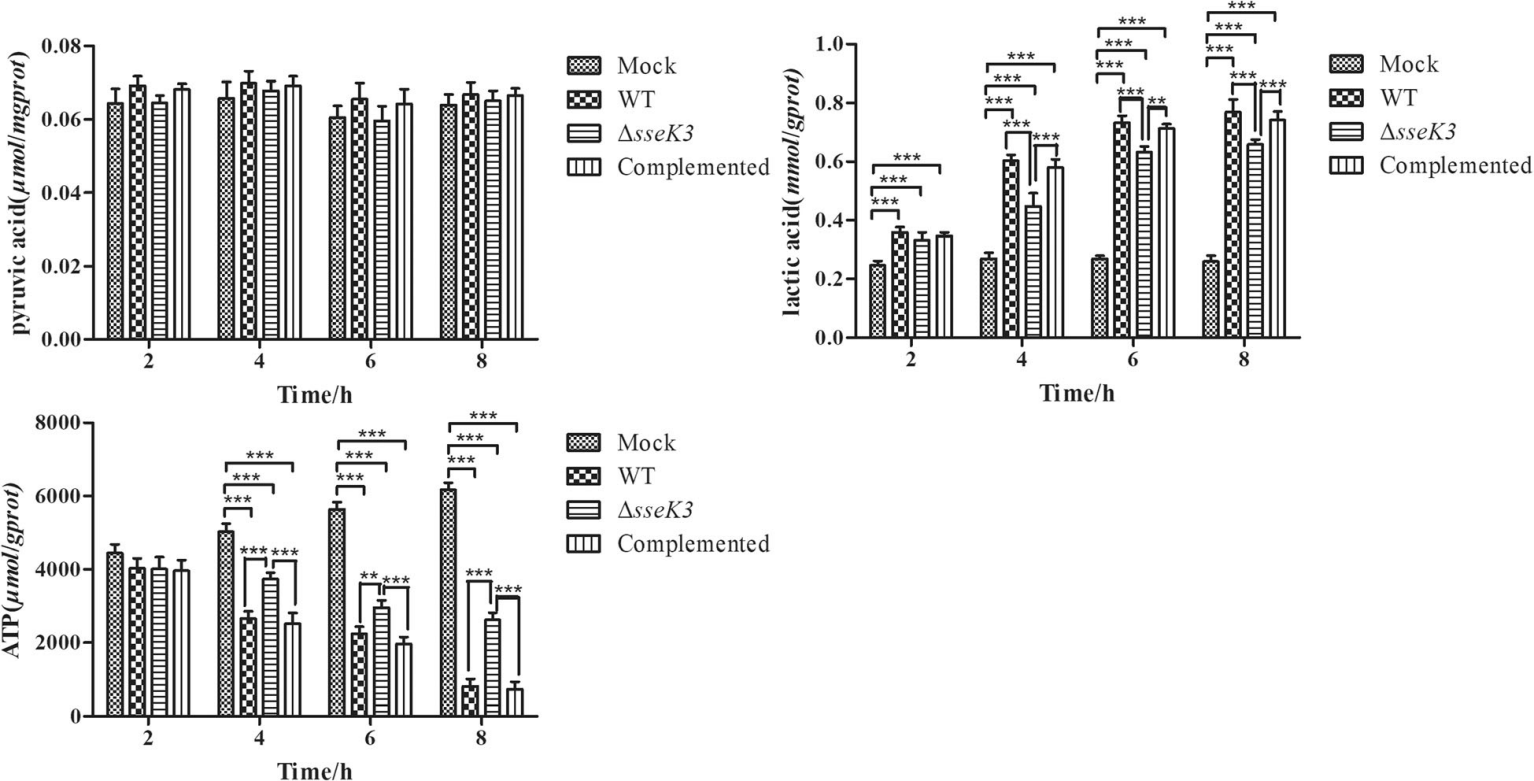
Glycolysis in RAW264.7 cells infected with WT, Δ*sseK3* mutant, or *sseK3-*complemented strains. The pyruvic acid, lactic acid, and ATP levels were measured by colorimetric assay and analyzed using two-way ANOVA with Bonferroni’s multiple-comparison test (*P < 0.05,**P < 0.01,***P < 0.001). Results are shown as mean ± SD

To determine whether SseK3 could affect glycolysis in macrophages infected with *S*. Typhimurium, pyruvic acid, lactic acid, and ATP levels were detected as previously described [21–23] (Fig. 3). The results showed that there were no significant differences in pyruvic acid levels between different groups at 2 h, 4 h, 6 h, and 8 h (*P* > 0.05). However, there were significant differences in lactic acid levels between the mock and infection groups at 2 h, 4 h, 6 h, and 8 h (*P* < 0.001). Macrophages glycolysis was significantly increased in the infection groups than in the mock group, and lactic acid levels were significantly lower in the Δ*sseK3* mutant group than in the WT and *sseK3*-complemented groups at 4 h, 6 h, and 8 h (*P* < 0.001). The investigations of glycolysis in macrophages suggested that SseK3 encoded by *sseK3* could boost lactic acid levels in macrophages. Further, ATP levels were significantly lower in infection groups than in the mock group at 4 h, 6 h, and 8 h (*P* < 0.001), and much higher in the Δ*sseK3* mutant group than in the WT and *sseK3*-complemented groups at 4 h, 6 h, and 8 h (*P* < 0.001). These results illustrated that SseK3 encoded by *sseK3* could improve glycolysis in macrophages by modulating lactic acid and the ATP levels.

## Discussion

Many *Salmonella* secreted proteins exert different virulence functions in the process of bacterial survival and replication in host cells [24]. In *Salmonella* SCVs T3SS2 helps *Salmonella* transport virulence (effector) proteins into host cells [25]. *S.* Typhimurium expresses the SseK family of proteins, including SseK1, SseK2 and SseK3, which are T3SS effectors [1, 26]. SseK3 is essential for cellular *Salmonella* infection, and plays a pivotal role in the natural host immune process [15, 17]. Several secretory proteins of *S.* Typhimurium contribute to the considerable stress observed during apoptosis [24, 25, 27, 28]. There were several studies about the function of SseK3. Joshua et al. found that SseK3 targeted death domain proteins in the TNF and TRAIL signaling pathways and employed the glycosyltransferase effectors to antagonize different components of death receptor signaling [29]. Yang et al. found that SseK3 could bind an E3 ubiquitin ligase (TRIM32) and influence NF-κB activity [15]. But Günster et al. found that SseK3 caused weak GlcNAcylation of TNFR1-associated death domain protein (TRADD) and inhibited TNF-α-induced apoptosis in *Salmonella*-infected macrophages [25]. These researches were contradictory. Nonetheless, there has been no research on the role of SseK3 in the relationship between apoptosis and glycolysis in *Salmonella*-infected macrophages. To our knowledge, this is the first study to determine the effect of *S.* Typhimurium SseK3 encoded by *sseK3* on macrophage glycolysis during *S.* Typhimurium-induced apoptosis.

Santo et al. found that early sipB-dependent and delayed sipB-independent mechanisms could cause the apoptosis induced by *S.* Typhimurium [30]. Further, *S.* Typhimurium without ribose chemoreceptors localized in tumor quiescence and induced apoptosis [31]. Moreover, research had shown that the cAMP receptor protein of *S.* Typhimurium could induce the macrophage apoptosis [32]. Based on previous functional studies of *Salmonella* genes in macrophages [15, 25, 33–35], we used the WT, Δ*sseK3* mutant, and *sseK3*-complemented strains to infect the macrophages to determine the relationship between *S.* Typhimurium *sseK3* and macrophages apoptosis. We found that the adherence and invasion percentages in the Δ*sseK3* mutant group were similar to those of WT group and *sseK3*-complemented group during the infecting period (*P* > 0.05), which confirmed that the WT, Δ*sseK3* mutant, and *sseK3*-complemented strains had the same level going through into the macrophages. Subsequently, the apoptosis in different infection groups were detected by the assay of flow cytometry. The percentage of apoptotic cells in the Δ*sseK3* mutant group (3.78%) was much lower than that in the WT (14.64%) and *sseK3*-complemented (11.81%) groups, indicating that deletion of the *sseK3* gene significantly affected host cell apoptosis.

The activation of caspases is essential for apoptosis induced by loss of the mitochondria membrane [36]. SopB of *Salmonella* could protect the host cell from caspase-3-induced apoptosis [37]. Activation of caspase-3 and caspase-9 indicated that apoptosis was activated in macrophages infected by *Escherichia coli* [38]. Further, caspase-8 influenced the synthesis of pro-IL-1β and was essential for apoptosis induced by *Salmonella* [39]. In this study, we found that caspase-3, caspase-8 and caspase-9 activity was significantly higher in the infection groups than in the mock group at 2 h, 4 h, 6 h, and 8 h (*P* < 0.001), indicating that apoptosis was activated in the infected macrophages. Furthermore, the caspase-3, caspase-8 and caspase-9 activity was much lower in the Δ*sseK3* mutant group than in the WT and *sseK3*-complemented groups, which suggested that SseK3 may be a pivotal component for *S.* Typhimurium activating apoptosis in macrophages. In our previous studies, we found that disruption of *sseK3* could reduce the virulence of *S.* Typhimurium (Du, et al. in press). We further speculated that the SseK3-mediated activation of apoptosis in macrophages may affect the virulence of *S.* Typhimurium.

Apoptosis and glycolysis are closely related. Comin-Anduix et al. found that fermented wheat germ extract (FWGE) inhibited glycolysis and induced apoptosis in tumor cells [40]. Jeong et al. found that the modification of glycolysis could change the sensitivity of apoptosis via the mitochondrial pathway [41]. Recently, research has shown that inhibition of glycolysis could block cell apoptosis [42]. Methyl jasmonate could inhibit the glycolysis and induce apoptosis of tumor cells [43], whereas propofol could improve glycolysis of cells and cause the apoptosis [44]. Pyruvic acid, lactic acid and ATP are important components in the process of cellular glycolysis. Various metabolic pathways produce pyruvic acid [45]. Pyruvic acid could enter the TCA cycle and be completely oxidized in aerobic conditions, or could become lactic acid under anaerobic conditions [46, 47]. HIF-1α could prevent the production of ATP and thus regulate the glycolysis of mouse granulosa cells [48]. We studied glycolysis in macrophages infected with WT, Δ*sseK3* mutant, or *sseK3*-complemented by detecting the levels of pyruvic acid, lactic acid, and ATP. There were no significant differences in intracellular pyruvic acid levels among the groups (*P* > 0.05). Compared to the WT and *sseK3*-complemented groups, the lactic acid levels of microphages infected by Δ*sseK3* mutant strain did not significantly change at 2 h (*P* > 0.05). However, there were significant differences between the WT and Δ*sseK3* mutant groups at 4 h (*P* < 0.001), 6 h (*P* < 0.001), and 8 h (*P* < 0.001), suggesting that the deletion of *sseK3* may reduce *S.* Typhimurium-induced glycolysis in macrophages, and SseK3 likely enhances glycolysis. Furthermore, lactic acid levels were significantly higher in the WT, Δ*sseK3* mutant, and *sseK3*-complemented strains, than in the mock group (*P* < 0.001), which illustrated that the anaerobic pathway was enhanced in macrophages of the infection groups. There was no significant difference in ATP levels among the groups at 2 h; however, the ATP levels were significantly higher in the Δ*sseK3* mutant group than in the WT and *sseK3*-complemented groups at 4 h (*P* < 0.001), 6 h (*P* < 0.001), and 8 h (*P* < 0.001), indicating that glycolysis was lower in the WT and *sseK3*-complemented groups than in the Δ*sseK3* mutant group, and SseK3 likely enhanced glycolysis. Moreover, the ATP levels of macrophages in the mock group were significantly higher than those in the infections groups at 4 h (*P* < 0.001), 6 h (*P* < 0.001), and 8 h (*P* < 0.001), which suggested that aerobic metabolism was predominant in the mock group, since less ATP is produced by glycolysis than by aerobic oxidation. Therefore, the deletion of *sseK3* likely decreased glycolysis in the Δ*sseK3* group compared with WT group, and SseK3 could improve glycolysis in macrophages infected by *S.* Typhimurium and thereby induce apoptosis. This conclusion was consistent with the results of Sumi et al’ [44] and Ding et al’ [32], showing that glycolysis and apoptosis of cells could be improved simultaneously. But it was not in agreement with the findings of Li et al’ [43] and Comin-Anduix et al’ [40], who showing that glycolysis of cells was inhibited and apoptosis of cells was enhanced, possible owing to differences in the type of cell. Glycolysis is the main source of energy in cancer cells, which use this metabolic pathway for ATP generation. Altered energy metabolism is a biochemical fingerprint of cancer cells, and is a “hallmark of cancer” [49]. Rosmarinic acid induces apoptosis in HepG2 cells, mainly via inactivation of the glycolytic pathway [50]. However, macrophages show a high level of plasticity, with the ability to undergo dynamic transition between M1 and M2 polarized phenotypes [51]. Lipopolysaccharide (LPS) of bacteria could induce RAW264.7 macrophages into the classical proinflammatory injury phenotype (M1) [52]. Activation of inflammatory responses could cause the apoptosis by mediating inactivation of the PI3K/Akt/GSK-3β signaling pathway [53]. Moreover, the proinflammatory stimulus, LPS, suppresses myelocytomatosis viral oncogene (Myc) expression and cell proliferation and engages a HIF1α-dependent transcriptional program responsible for heightened glycolysis [54]. We speculate that *S.* Typhimurium SseK3 might activate the M1 phenotype of macrophages and thereby activate HIF1-α, which enhances glycolysis and finally causes apoptosis. There could be several different signaling pathways involved in SseK3-mediated apoptosis and glycolysis of macrophages, which merit further study. We aim to further clarify the relationship between glycolysis and apoptosis in future studies.

## Conclusions

Our results cumulatively showed that SseK3 of *S.* Typhimurium could induce apoptosis and improve glycolysis in macrophages. Our findings may help to illustrate the mechanism by which *S.* Typhimurium induces macrophage apoptosis and provide a better understanding of the putative relationship between SseK3-induced macrophage apoptosis and glycolysis.

## Methods

### Bacterial strains, cells, and culture

*S.* Typhimurium SL1344, *S.* Typhimurium SL1344 ∆*sseK3* mutant (with deletion of *sseK3*), and *sseK3-*complemented bacterial strains used in this study were available in our laboratory. The ∆*sseK3* mutant was constructed using counter-selectable suicide vectors. The sseK3 gene was cloned into the pBR322 plasmid for complementation studies. RAW264.7 macrophage cells were obtained from the American type culture collection (ATCC, Manassas, VA), and cultured in Dulbecco’s modified Eagle medium (DMEM)/high-glucose medium (HyClone, USA) containing 10% fetal calf serum (FCS) in an incubator at 37 °C and 5% CO_2_.

### Adherence and invasion assay

Adhesion and invasion of RAW264.7 cells was assessed as previously described [55, 56]. A 24-well cell culture plate was inoculated with 1 × 10^5^ RAW264.7 cells per well. The WT, Δ*sseK3* mutant and *sseK3-*complemented strains were then added to RAW264.7 cells at a multiplicity of infection (MOI) of 100:1, with three replicate wells per strain. To allow complete contact between the bacteria and RAW264.7 cells, the plates were centrifuged at 1000 rpm and incubated in 5% CO_2_ for 2 h at 37 °C. For the adherence assay, the supernatants were aspirated, and the cells were washed three times with phosphate buffered saline (PBS). Subsequently, the cells were digested with 0.25% trypsin, plated in a gradient dilution and counted. For the invasion assay, the supernatants were aspirated, cells were washed three times with PBS, gentamicin-containing medium (100 μg/mL) was added, and the cells were incubated at 37 °C with 5% CO_2_. After incubation, the supernatants were aspirated, and the cells were washed three times with PBS. Subsequently, the cells were lysed using 0.1% Triton X-100, plated with a gradient dilution and counted.

### Flow cytometry assay

A 6-well cell culture plate was inoculated with 1 × 10^6^ RAW264.7 cells per well and incubated for 16 h. WT, Δ*sseK3* mutant and *sseK3-*complemented strains were incubated with RAW264.7 cells at a multiplicity of infection (MOI) of 100:1, with three replicate wells per strain. To allow complete contact between bacteria and RAW264.7 cells, the plates were centrifuged at 1000 rpm. Gentamicin-containing medium (100 μg/mL) was then added and the plates incubated at 37 °C with 5% CO_2_. After incubation, the supernatants were aspirated, and the cells were washed three times with PBS. The percentage of cells undergoing apoptosis was detected by flow cytometry using an Annexin V-FITC/PI apoptosis detection kit (KeyGEN BioTECH, Jiangsu, China). Cells from the infected and mock groups were digested with 0.25% trypsin, washed three times with ice-cold PBS, and suspended in 500 μL binding buffer. 5 μL Annexin V-FITC and 5 μL Propidium Iodide (PI) were added, and the solution was incubated in a dark room for 15 min at room temperature and immediately analyzed by flow cytometry (Beckman Coulter, Inc., Fullerton, CA, US).

### Caspase-3, caspase-8, and caspase-9 activity assay

Caspase-3, caspase-8, and caspase-9 activity was measured using a Caspase-3 Assay Kit, Caspase-8 Assay Kit, and Caspase-9 Assay Kit (Beyotime, Shanghai, China), respectively. A 6-well cell culture plate was inoculated with 1 × 10^6^ RAW264.7 cells per well and incubated for 16 h. WT, Δ*sseK3* mutant, and *sseK3-*complemented strains were incubated with the RAW264.7 cells at a MOI of 100:1, with three replicate wells per strain. The plates were centrifuged at 1000 rpm and gentamicin-containing medium (100 μg/mL) was added and incubated at 37 °C with 5% CO_2_. The supernatants were then aspirated, and the cells were washed three times with PBS. Subsequently, the cells of the infected and mock groups were digested by trypsinization without EDTA and washed three times with ice-cold lysis buffer, and treated with 100 μL lysis buffer on ice. After incubation for 15 min, the concentration of protein was detected using the Bradford protein assay kit (Beyotime, Shanghai, China). Subsequently, the cell lysates were incubated with Ac-DEVD-pNA for 4 h at 37 °C, the absorbance was read at 405 nm in a microplate spectrophotometer (Infinite 200 PRO NanoQuant, Tecan, Switzerland).

### Glycolysis assay

The glycolysis levels were measured using pyruvic acid, lactic acid, and ATP analysis kits, which were purchased from Nanjing Jiancheng Bioengineering Institute (Nanjing, China). WT, Δ*sseK3* mutant, and *sseK3-*complemented groups were treated as above similar methods. After incubation with gentamicin-containing medium (100 μg/mL), the supernatants were aspirated, and the cells were washed three times with PBS. Cells were then analyzed using the kits according to manufacturers’ instructions at 2 h, 4 h, 6 h, and 8 h. The protein concentration in each group was detected using the Bradford protein assay kit (Beyotime, Shanghai, China), and absorbance values for pyruvic acid analysis, lactic acid, and ATP analysis were read at 505 nm, 530 nm, and 636 nm, respectively in a microplate spectrophotometer (Infinite 200 PRO NanoQuant, Tecan, Switzerland).

### Statistical analysis

Data were presented as the mean ± standard deviation (SD) of three independent experiments. Two-way analysis of variance (ANOVA) with a *post-hoc* test (Bonferroni’s multiple-comparison test) was used to compare and assess statistical significance among all groups. *P* < 0.05 was considered statistically significant.

## Supplementary information

**Additional file 1: Figure S1.** Adherence and invasion assays for WT, Δ*sseK3* mutant, and *sseK3-*complemented strains in RAW264.7 cells. Bonferroni’s multiple-comparison test showed no significant differences between the groups (*P* > 0.05).

## Data Availability

The data generated and/or analyzed during the current study are available from the corresponding author on reasonable request.

## Abbreviations

SseK3: *S.* Typhimurium secreted effector K3
WT: Wild-type
HIF-1α: Hypoxia inducible factor-1α
SPI-2: Salmonella pathogenicity island 2
T3SS2: Type III secretion system 2
TRADD: TNFR1-associated death domain protein
FWGE: Fermented wheat germ extract
Myc: Myelocytomatosis viral oncogene
ATCC: American type culture collection
DMEM: Dulbecco’s modified Eagle medium
FCS: Fetal calf serum
PBS: Phosphate buffered saline
MOI: Multiplicity of infection
PI: Propidium Iodide
SD: Standard deviation
ANOVA: Analysis of variance
