# Supplementary figures and images for: Salmonella enterica serovar Typhimurium sseK3 induces apoptosis and enhances glycolysis in macrophages
Source: BMC Microbiol. 2020 Jun 9;20:151. doi: 10.1186/s12866-020-01838-z (PMC7282050; doi:10.1186/s12866-020-01838-z)

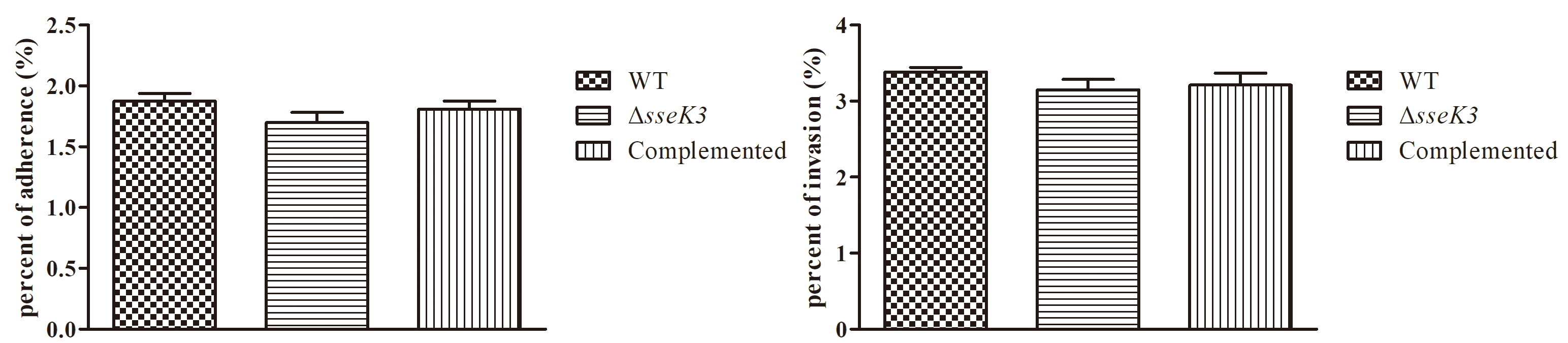

Supplement: Supplementary file 1 — Additional file 1: Figure S1. Adherence and invasion assays for WT, ΔsseK3 mutant, and sseK3-complemented strains in RAW264.7 cells. Bonferroni’s multiple-comparison test showed no significant differences between the groups (P > 0.05). [file 12866_2020_1838_MOESM1_ESM.tif]
